# Illuminating systematic differences in no job offers for STEM doctoral recipients

**DOI:** 10.1371/journal.pone.0231567

**Published:** 2020-04-29

**Authors:** Timothy J. Kinoshita, David B. Knight, Maura Borrego, Whitney E. Wall Bortz

**Affiliations:** 1 Engineering Education, Virginia Tech, Blacksburg, Virginia, United States of America; 2 Mechanical Engineering, University of Texas at Austin, Austin, Texas, United States of America; Iowa State University, UNITED STATES

## Abstract

This study examines differences across demographic subgroups in the phenomenon of recent doctoral recipients seeking work but having no job offers for employment. Gender and race/ethnicity have been identified as two characteristics with considerable issues of representation in a number of science and engineering fields, particularly at the doctoral level. Using the NSF Survey of Earned Doctorates dataset, which includes over 298,000 respondents in the biological sciences, engineering, and physical sciences since 1977, we use logistic regression modelling to examine the likelihood of doctoral recipients having no offers at the time of graduation as a function of race, gender, family and funding variables. We find that across the fields of biology, engineering, and physical sciences, women and underrepresented minorities have a higher prevalence of having no job offers, but this relationship has notable interaction effects for family variables and doctoral program funding mechanism. Importantly, marital status accounts for differences in job offers between genders that deserves further exploration.

## Introduction

Underrepresentation of women and individuals from minoritized racial/ethnic groups within the science, technology, engineering, and mathematics (STEM) fields is a persistent issue. Despite increased participation at every educational stage [1], both women and underrepresented minorities (URMs) earn lower salaries and receive fewer promotions than their well-represented counterparts. Such disparities have inspired a wealth of research into the underlying factors behind this underrepresentation.

In addition to graduate education research focused on equity and inclusion issues, the increased prevalence of non-academic career pathways for doctoral recipients has inspired closer examination of the diversity of career trajectories [2]. Although the preparation of future college and university faculty members remains one of the primary functions of doctoral education [3], recent efforts have increasingly sought to define and explain the increasing variety of career pathways for doctoral recipients [4]. Prior research has identified shifts in non-academic doctoral employment (e.g. [5,6]), influences underlying non-academic job interest [7], and strategies that doctoral students employ to navigate the non-academic job market [8]. Inspired by these research strands, this work seeks to advance greater understanding of doctoral career outcomes, following the recommendations of June [9].

Several national organizations have begun coordinated efforts to inform the current state of doctoral careers. In 2011, the Council of Graduate Schools in collaboration with higher education leaders from 16 countries released a statement of principles to establish a clearer understanding of graduate student careers [10]. One of the stated principles of this collaboration is to provide prospective students with knowledge of the wide array of doctoral career paths. A number of associations also have been created with the aim of providing increased transparency regarding graduate education outcomes. Organizations such as the Broadening Experiences in Scientific Training (BEST) consortium and the Coalition for Next Generation Life Science [11,12] seek to share data and best practices regarding varied graduate career pathways (e.g., non-tenure-track academic, industry, and government positions).

This research paper complements such efforts by investigating the prevalence of doctoral recipients seeking work, but having no offers for employment at the time of graduation and examining differences across demographic subgroups, specifically gender and race/ethnicity. Gould [12] identified that with the rapid rise in the number of students graduating with doctorates, the proportion of doctoral recipients graduating with no commitment to employment or postdoctoral study was on the rise, most noticeably since 2003. Although no study that we identified has sought to examine discrepancies in no job offers across gender and race/ethnicity, prior research has identified notable underrepresentation of women and URM doctorate holders at all stages of the tenure-track faculty pathway [13], especially in science and engineering fields. Additional research efforts have focused on understanding attempts to improve the hiring [14,15], retention, and promotion [16] of members from these groups.

We acknowledge that PhD holders have the lowest unemployment rate by educational attainment at 1.5%, as compared to 2.2% and 2.5% for Master’s and Bachelor’s holders, respectively [17]. The primary phenomenon of interest for this study, however, is immediate job offers at the time of PhD completion and whether there are systematic differences across subpopulations. As PhDs are some of the most academically trained job seekers in the labor market, eventually finding work is of lower concern. The driving assumption is that a lack of immediate job offers, with specific focus on recent trends and disproportionate impacts across subpopulations, are indications of an inequitable education-workforce system that deserves attention.

Our study addresses the following research questions:

1. How has the rate of doctorate recipients with no offers immediately following graduate school changed over the past 40 years for science and engineering fields across demographic subgroups?
2. What are some of the factors related to differences in no offers by gender and race/ethnicity?

## Literature review

Gender and race/ethnicity have been identified as two characteristics with considerable issues of representation in a number of science and engineering fields, particularly at the doctoral level. Although women have made gains in some fields, such as in biological sciences where they earned half of doctoral degrees in 2015, they earned less than one-third of the doctorates awarded in mathematics, computer sciences, and engineering [18]. Similarly, Black, Hispanic, and American Indian and Alaskan Native students earned only 14 percent of all U.S. science and engineering doctoral degrees in 2015, despite comprising nearly one-third of the U.S. population [19].

Even after earning the PhD, these patterns of underrepresentation extend to career outcomes with women and underrepresented minority men being less likely than white men to obtain academic positions at research universities and more likely to be in part-time or non-tenure track positions [13]. A substantial body of research has examined post-PhD employment, with consideration for differences by gender and race (note: throughout this manuscript, we refer to immediate job placement for doctoral recipients as post-PhD employment, in contrast to postdoctoral appointments. Although postdocs are considered in our analysis, they are not the only employment outcome studied). Although much of this research has focused on academic career outcomes, theoretical contributions on employment choices and hiring practices within the academic sector can provide a meaningful basis for a discussion on post-PhD employment in general.

Our review and analysis also focus on two additional categories of variables: family (i.e., marital status and dependents), and doctoral student funding mechanisms. As we elaborate later in this section, some of the previously observed differences in career outcomes for men and women can be explained by marriage or having dependent children.

We also seek to gain a greater understanding of doctoral education by connecting career outcomes to doctoral experiences. Prior research has identified that the experiences and professional relationships afforded through different funding opportunities have a relationship with PhD completion [20], productivity [21], and career outcomes [22]. Because of its varied role in facilitating researcher career and professional development and its role as a programmatic mechanism over which academic departments and funding agencies have some control, we include the primary funding mechanism as a variable in our analysis. Furthermore, because fellowships are used as targeted recruiting mechanisms for both women and URM doctoral students [22], a greater understanding of funding mechanism as a moderating influence on employment outcomes is warranted.

### Differences by gender

Within many science and engineering fields, women are underrepresented in college majors, graduate school programs, and tenure-track academic positions [23]. Scholars attempting to explain these disparities have taken either a supply- or demand-side approach. The supply side approach often uses a “leaky pipeline” metaphor to suggest that gender disparities result from a shortage of women seeking educational credentials and employment opportunities because of cultural and structural barriers [24]. Recently, increasing attention has focused on demand-side processes that include institutional mechanisms that promote or hinder the successful recruitment, retention, and advancement of women [14], including family friendly employment policies and workplace biases.

Research also has examined biases in hiring practices that differentiate by gender, and the results are somewhat mixed. Numerous small-scale experiments find bias by interviewers and evaluators against hypothetical female applicants and their work products relative to hypothetical male applicants with the same credentials (e.g. [25]). Similarly, prior research has suggested that academic scientists express implicit biases that reflect cultural stereotypes that emphasize male scientific competence [26]. A more recent and comprehensive examination of hiring biases may suggest that the situation may be improving. A national randomized experiment on 873 tenure-track faculty from biology, engineering, economics, and psychology at 371 institutions found a two-to-one preference for women by reviewers of both genders across both math- and non-math-intensive fields [27].

### Differences by race

Similar to women, racial and ethnic URM students and graduates are underrepresented at all key transition points along the STEM career pipeline with a cumulative impact at later stages [28]. Also, similar to women, URM students and graduates can experience implicit or explicit biases in the evaluation of their work [29]; a values misalignment with certain types of faculty work [30] contributes to these disparities. Gibbs et al. [31] found that URM men and women in the biomedical sciences reported lower levels of interest in faculty positions at research-intensive universities, even after controlling for career interests at the time of PhD entry, scholarly productivity, faculty mentorship, and research self-efficacy. URM and well-represented doctoral recipients were also found to differ on the influence of prestige, salary, family influence, and faculty advisor influence on their post-PhD employment. We seek to examine whether we observe different employment outcomes across racial/ethnic groups, which may manifest from different career preferences and tendencies away from faculty work.

### Influence of family

Work-family balance has been shown to play an important role in the employment outcomes for doctoral recipients, with a pronounced effect for women. Prior research in the context of the traditional American heterosexual family structure finds that women tend to spend more time on parenting and housework-related responsibilities than their male partners, even when both individuals hold full-time jobs [32]. Workplace structures that are not, or even appear to be not, conducive to alignment with work-family balance can push graduate students, especially female students, away from certain career paths [33]. Perna [34] and Long [35] both found that marriage increases women PhD holder’s likelihood of part-time, non-tenure-track employment and spending time out of the workforce. Prior research has also found that the unequal socioeconomic structure of men and women in heterosexual relationships often places the man’s career at a higher priority than the woman’s. Harper et al. [36] found that women were more likely than men to leave a faculty position to accommodate a spouse’s career. Much of the research on the moderating effect of family on doctoral employment outcomes focuses on gendered effects. The limited research on racial influences has found that gender inequalities persist across racial/ethnic and socioeconomic lines.

### Influence of graduate funding

Blume-Kohout & Adhikari [22] examined doctoral recipients in the biomedical sciences and assessed the likelihood of their transition into scientific research-focused employment as a function of their primary funding mechanism. They found that students primarily supported as research assistants had a 4.6 to 11 percentage point higher probability of taking a research and development job in comparison with those on fellowships. Even though their focus was on a specific post-PhD employment outcome, Blume-Kohout and Adhikari’s examination is valuable for our study; doctoral students’ primary funding mechanism can have an influence on immediate career outcomes. Although the financial subsidies across different funding mechanisms may be similar, the incentives that each create for graduate students’ interaction with faculty members and skill development may differ considerably. Research assistantships, which are particularly prevalent in the STEM fields [18], are designed for students to gain exposure to research and benefit from supervision and interaction with senior researchers [37]. In addition, because research assistants’ work contributes directly to the faculty members’ professional output, faculty members have incentives to train and actively manage their research assistants, especially in comparison to students supported via externally funded fellowships. These different scenarios can lead to tangible differences in output; students funded via graduate research assistantships (GRAs) have higher publishing rates than students on graduate teaching assistantships (GTAs) [38], a metric that has been tied to increased probability of employment in academic, research-oriented positions [31]. In contrast, fellowships can afford a greater independence for students to pursue their own research interests but can present barriers to connecting with research labs [39].

## Methods

### Data source

This analysis focuses on the prevalence and predictors of no job offers at the time of PhD completion for students in biology, engineering, and physical sciences. Data were drawn from the National Science Foundation’s Survey of Earned Doctorates (SED) data set. The SED is characterized by comprehensive coverage of doctoral recipients from institutions in the United States. Using a combination of self-administered paper surveys, web-based surveys, and computer-assisted telephone interviews, graduate schools typically require SED responses at the time of degree completion. In FY17, 91.4% of the 54,664 individuals who were granted a research doctorate completed the SED [40]. This research was approved by the Virginia Tech Institutional Review Board (15–601), FWA00000572. All analyses were conducted on de-identified secondary data.

SED variables include individuals’ characteristics and pre-doctorate educational history, funding received during graduate school, and post-graduate school plans. The period of record for this analysis begins with students graduating in fiscal year 1977, as some variables of interest were not collected prior to this year, and ends in fiscal year 2016 (i.e., July 1, 2015 to June 30, 2016).

The dependent variable for this analysis, no job offers, was derived from a closed-ended survey item asking the status of post-graduation plans with 7 options. We converted this item into a binary variable by isolating responses to one specific option: “Seeking position but have no specific prospects.” This option was one of seven options that also included: “Returning to, or continuing in, predoctoral employment”; “Having signed contract or made definite commitment for a ‘postdoc’ or other work”; “Negotiating with one or more specific organizations”; “Other full-time degree program”; “Do not plan to work or study”; and “Other”. Our focus for this study (i.e., seeking position but have no specific offers) sits in contrast to these other career outcome options that imply receipt of a job offer with a specific employer.

Starting with the 2001 survey administration, the post-graduation survey item was altered into two separate questions with a logical skip separating questions into items regarding definite commitments and specific post-doctoral plans. We conducted a check on the soundness of using data across the full period of record (i.e., 1977 to 2016), which we describe more fully in the subsequent robustness checks section.

We limited our analyzed population to U.S. citizens and permanent residents, representing 64 percent of the total available population under the period of study. We chose to withhold temporary residents from this analysis because the job search process comes with different intentions and limitations for international students in comparison to domestic students. We acknowledge that international students make up a valuable and non-negligible population within doctoral education, especially within the STEM fields, and future research will seek to incorporate this group of doctoral recipients.

Our usage of the gender variable is taken from the SED item which asks respondents to report as male or female. In alignment with previous research, we interpret this response as the social construct of gender. We acknowledge that this construct is not binary, but given limitations in how the data were collected, we analyze and interpret utilizing these two values.

Additionally, we filtered our sample to four primary racial categories: Asian, Black, Hispanic, and White. These categories represented 96 percent of the total sample. The American Indian or Alaskan Native category, an underrepresented population in higher education, constituted less than one percent of the sample and was not included in this analysis because of the ambiguity of drawing conclusions from a small sample size. Respondents reporting other racial categories that included “Multiple Racial Responses,” “Other,” “Unknown,” and “Refused to Respond” also were removed because of the ambiguity of interpretation.

We present summary statistics for all predictor and control variables disaggregated by academic field in Table 1. In addition, frequencies and no job offer outcome percentages for variables cross tabulated by gender and race for the full period of record and disaggregated by academic field are presented in Tables 2 and 3. Academic fields include all subfields within biology/biomedical sciences, engineering, and physical sciences. We chose to include subfields within computer and information sciences within engineering to acknowledge their inclusion within colleges of engineering at some institutions. Table A1 in S1 Appendix displays the subfields within each field category as of the 2016 SED administration.

**Table 1:** Summary statistics by field.

		BIOLOGY		ENGINEERING		PHYSICAL SCIENCES	
		n	%	n	%	n	%
GENDER							
	Male	65070	55.7%	73199	82.3%	59647	76.2%
	Female	51824	44.3%	15750	17.7%	18670	23.8%
RACE							
	White	95545	81.7%	67248	75.6%	67304	85.9%
	Asian	12582	10.8%	15566	17.5%	6778	8.7%
	Black	3486	3.0%	2662	3.0%	1583	2.0%
	Hispanic	5281	4.5%	3473	3.9%	2652	3.4%
FUNDING							
	GRA	41647	35.6%	40420	45.4%	39196	50.0%
	Employer	1446	1.2%	6520	7.3%	1275	1.6%
	Fellowship	45265	38.7%	21677	24.4%	15156	19.4%
	Personal	13269	11.4%	13413	15.1%	6605	8.4%
	GTA	14967	12.8%	6919	7.8%	16085	20.5%
MARITAL							
	Not Married	60262	51.6%	39315	44.2%	42113	53.8%
	Married	56632	48.4%	49634	55.8%	36204	46.2%
Dependents							
	0	89335	76.4%	60903	68.5%	61558	78.6%
	1	16006	13.7%	13068	14.7%	9565	12.2%
	2	8623	7.4%	9962	11.2%	5173	6.6%
	3+	2930	2.5%	5016	5.6%	2021	2.6%
TOTAL		116894	100.0%	88949	100.0%	78317	100.0%

**Table 2:** Summary statistics for each field by gender.

		MALE		FEMALE		TOTAL	
		n	% No Offers	n	% No Offers	n	% No Offers
BIOLOGICAL SCIENCES							
TOTAL		65070	14.8%	51824	19.6%	116894	16.9%
RACE							
	White	54884	14.3%	40661	18.1%	95545	15.9%
	Asian	6208	17.8%	6374	25.9%	12582	21.9%
	Black	1414	19.5%	2072	25.4%	3486	23.0%
	Hispanic	2564	16.5%	2717	23.5%	5281	20.1%
FUNDING							
	GRA	24166	13.8%	17481	18.3%	41647	15.7%
	Employer	965	8.9%	481	19.1%	1446	12.3%
	Fellowship	22950	13.6%	22315	19.3%	45265	16.4%
	Personal	8116	15.2%	5153	20.1%	13269	17.1%
	GTA	8873	20.9%	6094	25.1%	14967	22.6%
MARITAL							
	Not Married	31051	16.7%	29211	18.4%	60262	17.5%
	Married	34019	13.1%	22613	21.2%	56632	16.3%
Dependents							
	0	47069	15.5%	42266	19.1%	89335	17.2%
	1	9745	13.5%	6261	21.8%	16006	16.7%
	2	6026	12.2%	2597	21.2%	8623	14.9%
	3+	2230	13.3%	700	22.3%	2930	15.5%
ENGINEERING							
TOTAL		73199	15.6%	15750	21.6%	88949	16.7%
RACE							
	White	56414	13.9%	10834	18.9%	67248	14.7%
	Asian	12319	21.4%	3247	28.6%	15566	22.9%
	Black	1824	23.0%	838	24.5%	2662	23.4%
	Hispanic	2642	18.9%	831	26.2%	3473	20.6%
FUNDING							
	GRA	33994	16.7%	6426	23.3%	40420	17.8%
	Employer	5794	4.9%	726	5.8%	6520	5.0%
	Fellowship	15908	13.8%	5769	21.3%	21677	15.8%
	Personal	11679	16.4%	1734	19.6%	13413	16.8%
	GTA	5824	23.1%	1095	26.9%	6919	23.7%
MARITAL							
	Not Married	31295	19.2%	8020	20.0%	39315	19.3%
	Married	41904	12.9%	7730	23.3%	49634	14.5%
Dependents							
	0	48590	17.3%	12313	21.3%	60903	18.1%
	1	11061	13.8%	2007	23.1%	13068	15.2%
	2	8889	11.0%	1073	22.3%	9962	12.3%
	3+	4659	10.4%	357	23.0%	5016	11.3%
PHYSICAL SCIENCES							
TOTAL		59647	15.7%	18670	19.3%	78317	16.6%
RACE							
	White	52197	14.7%	15107	17.5%	67304	15.3%
	Asian	4689	23.4%	2089	28.1%	6778	24.8%
	Black	993	23.4%	590	26.4%	1583	24.5%
	Hispanic	1768	21.4%	884	24.2%	2652	22.4%
FUNDING							
	GRA	30556	14.7%	8640	18.2%	39196	15.5%
	Employer	1051	3.7%	224	4.5%	1275	3.8%
	Fellowship	10349	12.2%	4807	16.5%	15156	13.6%
	Personal	5411	15.4%	1194	20.0%	6605	16.3%
	GTA	12280	22.3%	3805	26.2%	16085	23.2%
MARITAL						
	Not Married	30870	17.9%	11243	17.7%	42113	17.8%
	Married	28777	13.4%	7427	21.8%	36204	15.1%
Dependents							
	0	45515	16.5%	16043	18.7%	61558	17.1%
	1	7860	13.8%	1705	23.0%	9565	15.5%
	2	4459	13.0%	714	23.0%	5173	14.4%
	3+	1813	11.3%	208	25.5%	2021	12.7%

**Table 3:** Summary statistics for each field by race.

		WHITE		ASIAN		BLACK		HISPANIC	
		n	% No Pros	n	% No Pros	n	% No Pros	n	% No Pros
BIOLOGICAL SCIENCES									
TOTAL		95545	15.9%	12582	21.9%	3486	23.0%	5281	20.1%
GENDER									
Male		54884	14.3%	6208	17.8%	1414	19.5%	2564	16.5%
Female		40661	18.1%	6374	25.9%	2072	25.4%	2717	23.5%
FUNDING									
	GRA	34803	14.3%	4832	23.2%	730	22.3%	1282	21.2%
	Employer	1408	9.2%	219	12.3%	50	14.0%	69	20.3%
	Fellowship	34546	15.4%	5575	19.7%	2077	21.0%	3067	18.3%
	Personal	11668	16.3%	838	21.8%	341	29.0%	422	20.9%
	GTA	13120	21.6%	1118	28.9%	288	33.7%	441	29.0%
MARITAL									
	Not Married	49345	16.7%	6060	20.2%	2123	24.0%	2734	20.4%
	Married	46200	15.0%	6522	23.4%	1363	21.5%	2547	19.8%
Dependents									
	0	73388	16.3%	9593	21.2%	2430	22.6%	3924	20.1%
	1	12757	15.1%	1938	24.0%	537	23.6%	774	20.7%
	2	7010	13.2%	866	25.2%	337	21.4%	410	18.5%
	3+	2390	13.8%	185	18.9%	182	29.1%	173	20.8%
ENGINEERING									
TOTAL		67248	14.7%	15566	22.9%	2662	23.4%	3473	20.6%
GENDER									
Male		56414	13.9%	12319	21.4%	1824	23.0%	2642	18.9%
Female		10834	18.9%	2089	28.1%	838	24.5%	831	26.2%
FUNDING									
	GRA	29896	15.8%	8610	23.3%	688	26.0%	1226	21.9%
	Employer	5193	4.2%	835	8.4%	217	8.3%	275	6.2%
	Fellowship	16480	14.1%	2687	20.1%	1210	23.0%	1300	21.8%
	Personal	10688	14.5%	1906	26.9%	381	26.5%	438	20.3%
	GTA	4991	21.9%	1528	28.9%	166	28.9%	234	25.6%
MARITAL									
	Not Married	30562	18.0%	5798	23.3%	1421	25.7%	1534	24.4%
	Married	36686	12.0%	9768	22.7%	1241	20.9%	1939	17.6%
Dependents									
	0	46961	16.6%	9960	23.6%	1663	23.6%	2319	22.7%
	1	8868	11.8%	3285	22.7%	393	24.7%	522	18.6%
	2	7340	10.0%	1853	18.9%	355	20.3%	414	15.2%
	3+	4079	8.7%	468	25.2%	250	25.2%	218	13.8%
PHYSICAL SCIENCES									
TOTAL		67304	15.3%	6778	24.8%	1583	24.5%	2652	22.4%
GENDER									
Male		52197	14.7%	4689	23.4%	993	23.4%	1768	21.4%
Female		15107	17.5%	2089	28.1%	590	26.4%	884	24.2%
FUNDING									
	GRA	33971	14.3%	3783	23.2%	459	25.1%	983	22.1%
	Employer	1089	3.1%	96	8.3%	45	4.4%	45	11.1%
	Fellowship	12609	12.3%	1037	20.5%	611	21.1%	599	28.2%
	Personal	5995	15.6%	301	26.6%	115	16.5%	194	21.6%
	GTA	13640	21.6%	1561	32.4%	353	34.8%	531	30.1%
MARITAL									
	Not Married	36831	17.2%	2985	21.7%	931	24.8%	1366	22.3%
	Married	30473	13.1%	3793	27.3%	652	24.1%	1286	22.4%
Dependents									
	0	53592	16.1%	4892	24.2%	1091	23.4%	1983	23.0%
	1	7695	12.7%	1274	27.9%	229	27.5%	367	21.8%
	2	4291	12.7%	502	23.7%	163	23.3%	217	18.9%
	3+	2026	9.1%	110	23.6%	100	32.0%	85	17.6%

### Analytical method

**Fig 1:**
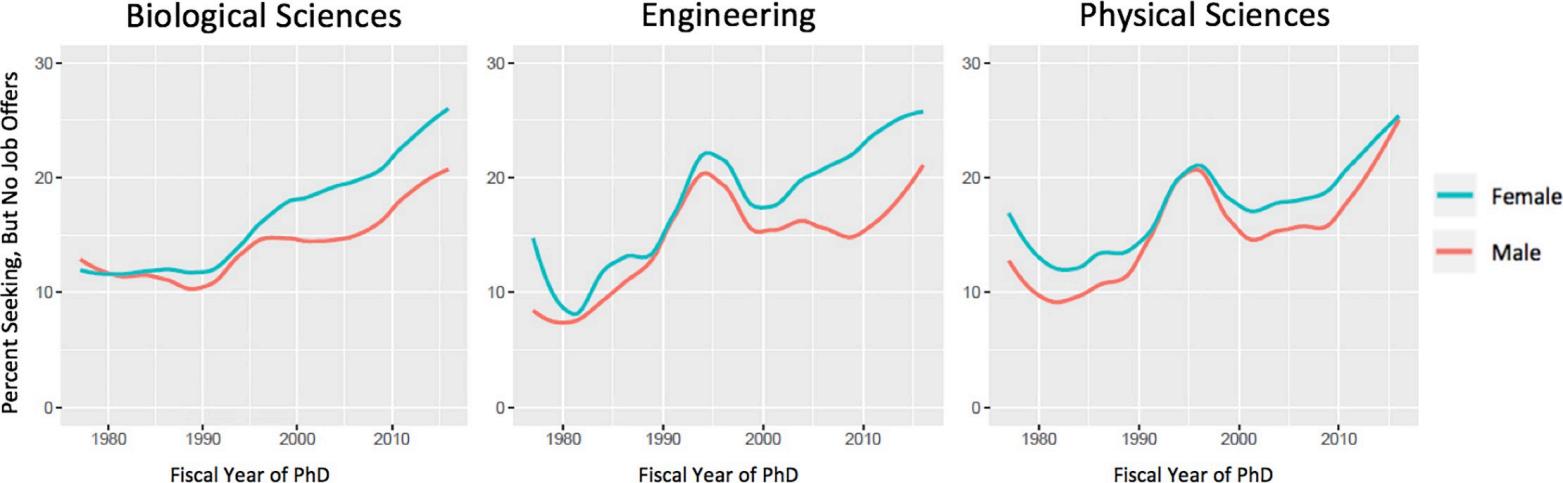
Percent seeking, but no job offers from 1977 to 2016 by gender.

**Fig 2:**
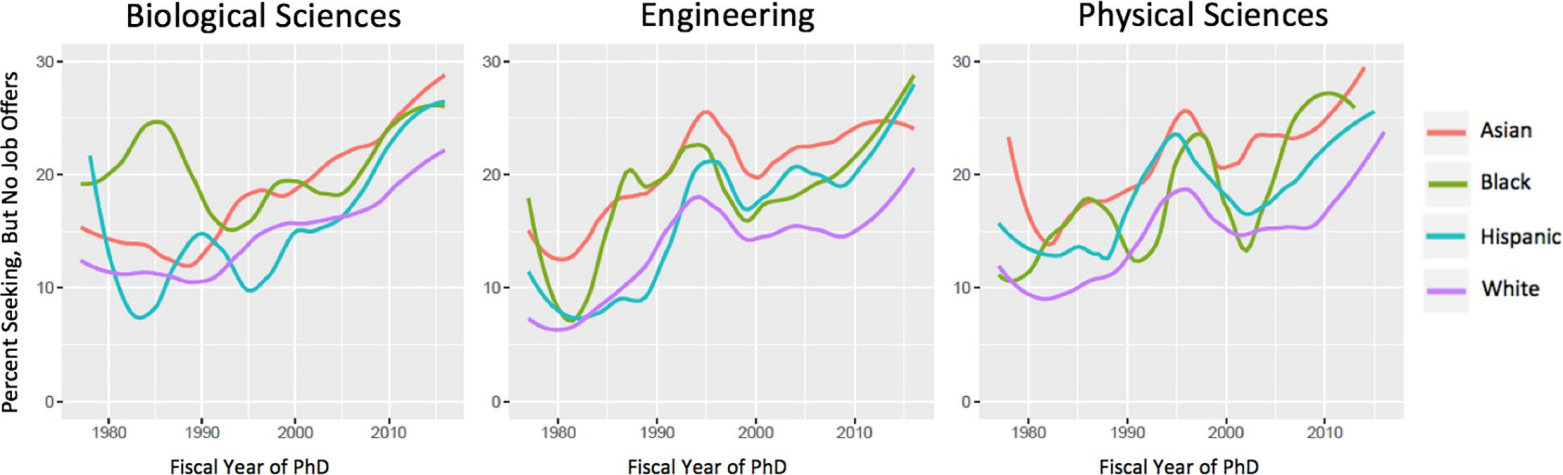
Percent seeking, but no job offers from 1977 to 2016 by race.

To investigate the relationship between the predictor variables and our outcome variable of interest (i.e., no job offers), we modeled a series of three binomial logistic regressions separately for each of three field categories: 1) biological sciences, 2) engineering, and 3) physical sciences. Within each field category, we modeled the main effects of gender and race, in addition to variables relating to family and funding mechanism as control variables. As represented in Figs 1 and 2, there is marked variation over time in the annual percentage of doctoral earners reporting no job offers at the time of graduation. To account for this, we have included a fixed effect for year of graduation.

In addition, we chose to interpret the models’ primary main effects using an institution fixed-effects model because of our interest in differences across demographic subpopulations. This approach focuses on comparisons that highlight demographic differences occurring within the same field within an institution. Also relevant to our outcome variable (i.e., the presence of a job offer at graduation), institution- and program-level reputations have been found to influence post-doctoral career outcomes—our modelling approach does not consider that effect. To include this fixed effect, we utilized a filter to include survey respondents from institutions with at least 30 respondents.

**Fig 3:**
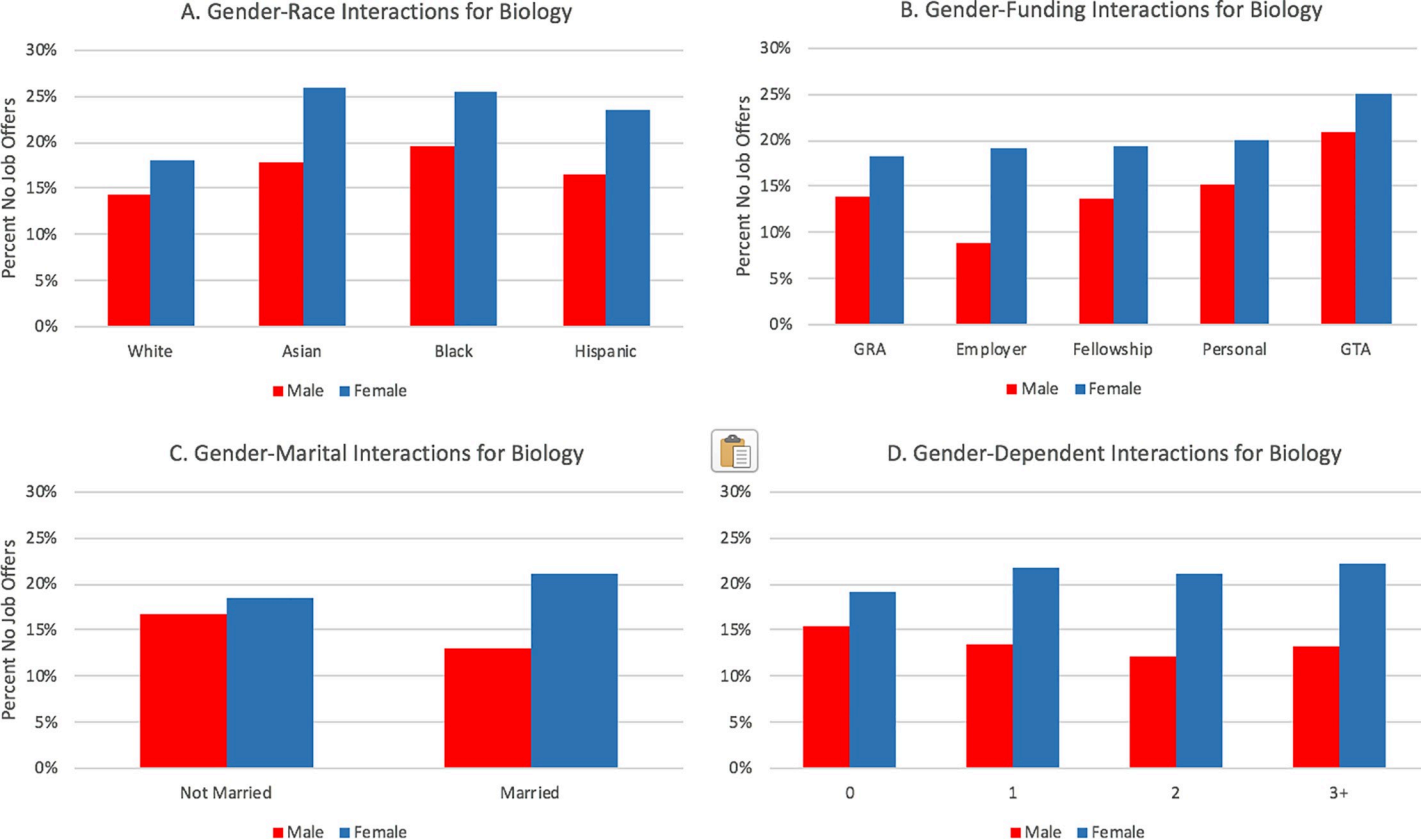
Interaction plots of gender interactions for biological sciences.

Table 4 displays coefficients and standard errors for the logistic regression for the three field categories, as well as the associated significant odds ratios, which includes all main effect and control variables and year and institution fixed-effects. Finally, in a separate model, we modeled interaction effects between gender and race and the family and funding variables, controlling for the main effects in Table 4. We visually display interaction effects in Figs 3 through 8 in the Results section; coefficients of the expanded model with interaction effects included can be found in the Appendix (Tables B1, B2, and B3 in S2 Appendix; note: coefficients for main effects differ from Table 4 because of the inclusion of interaction effects).

**Table 4:** Logistic regression coefficients, standard errors, and odds ratios of gender, race, funding, and family main effects for biological sciences, engineering, and physical sciences. Dependent variable: Seeking, but No Job Offer.

		BIOLOGICAL SCIENCES		ENGINEERING		PHYSICAL SCIENCES	
	Intercept	-2.728 *** (0.133)		-2.417 *** (0.233)		-9.837 (72.46)	
GENDER (Ref: Male)							
	Female	0.224 *** (0.016)	1.25	0.256 *** (0.023)	1.29	0.085 *** (0.023)	1.09
RACE (Ref: White)							
	Asian	0.336 *** (0.025)	1.40	0.493 *** (0.024)	1.64	0.475 *** (0.033)	1.61
	Black	0.318 *** (0.047)	1.37	0.395 *** (0.051)	1.48	0.287 *** (0.068)	1.33
	Hispanic	0.142 *** (0.037)	1.15	0.241 *** (0.047)	1.27	0.266 *** (0.052)	1.30
FUNDING (R: Research Asst.)							
	Employer	-0.860 *** (0.084)	0.42	-1.489 *** (0.063)	0.23	-2.048 *** (0.158)	0.13
	Fellowship	-0.112 *** (0.021)	0.89	-0.197 *** (0.024)	0.82	-0.258 *** (0.030)	0.77
	Personal	0.106 *** (0.030)	1.11	-0.095 ** (0.032)	0.91	-0.094 * (0.041)	0.91
	Teaching Asst.	0.390 *** (0.026)	1.48	0.263 *** (0.033)	1.30	0.350 *** (0.025)	1.42
FAMILY							
	Married (R: Not Marr)	-0.093 *** (0.018)	0.91	-0.314 *** (0.021)	0.73	-0.238 *** (0.023)	0.79
	Dependents (cont.)	-0.086 *** (0.012)	0.92	-0.120 *** (0.013)	0.89	-0.138 *** (0.016)	0.87

* p<0.1

** p<0.01; p<0.001

^ Control variables are Age at Doctorate, Father’s Education, Mother’s Education, and Time to Degree

### Robustness checks

To account for potential sources of bias in survey responses and across field differences, we also investigated three additional sets of models. In the first model, we filtered the SED data to only include responses starting in Fiscal Year 2001. Beginning with that year’s administration, the post-graduation survey item was altered into two separate questions with a logical skip separating questions into items regarding definite commitments and specific post-doctoral plans. We modeled these data using the same variables as in the previous model. Results are presented in Table B4 in S2 Appendix.

We also conducted a robustness check to account for spurious cross-field comparisons through a model that included all three disciplines. This model included main effects and controls, in addition to field-level interaction effects with the four primary variables of interest (i.e., gender, race, family, and funding). Results are found in Table B5 in S2 Appendix.

Finally, recognizing the limitations regarding use of the SED to examine post-doctoral career outcomes, we sought to conduct a high-level check on the persistence of our identified patterns using the follow-up, longitudinal, sample-based Survey of Doctoral Recipients (SDR). Using linked data on nearly 20,000 respondents across the biological sciences, engineering, and the physical sciences, we examined SED respondents who initially reported that they were seeking work but had no job offers compared to their employment status within 3 years of doctorate recipient. We present the results of these analyses in Table B6 in S2 Appendix.

Additionally, we utilized survey items that are unique to the SDR to examine employment patterns more thoroughly. Using the item, “To what extent was your work on your principal job related to your first U.S. doctoral degree” and “Did these factors influence your decision to work in an area outside the field of your first U.S. doctoral degree,” we examined each factor by gender and marital status for SED respondents who initially reported that they were seeking work but had no job offers. We report frequencies and percentages in Table B7 in S2 Appendix.

### Limitations

This study explores the phenomenon of recent doctoral recipients who are seeking but unable to obtain a specific job offer at the time of survey completion (typically timed near their graduation date). In this analysis, our interpretation is limited to the existing format of the SED survey item that is worded as “seeking position, but have no specific prospects.” We acknowledge that survey respondents could interpret and respond to this option in a number of ways. In comparison to the other question items within that option, we assert that our short-hand interpretation of “seeking, but no job offer” is a fair one; however, the reader should be aware of this interpretation.

Existing research on doctoral career choice has also focused on other experiences during the doctoral program and their relationship with career intentions (e.g., [41]). Data limitations within the SED prevent the inclusion of any variables related to career intentions or motivations. Additionally, the SED has been criticized as an inadequate measure of employment status because of the variable timing of its administration across institutions [42]. However, we would not expect the timing of the survey administration to yield different results across subpopulations within the same institution. Finally, we acknowledge how our decisions with respect to aggregating subpopulations may influence results—for example, we intentionally did not include international students, and we grouped Asian students as a single category, which could mask some differences within this group [43].

## Results

Over the 40-year time frame examined, each field has experienced an increase in the percentage of doctoral recipients who were seeking but found no job offers at the time of completing the survey (i.e., generally at the end of a student’s PhD program) (Fig 1 and Fig 2). The steepness of the curves in the most recent 15 years demonstrates that a greater percentage of PhD recipients have been graduating without job offers. Over this same time period, the overall number of PhD recipients has been increasing; coupled with these figures, it appears as if the number of doctoral recipients is outpacing the job market for PhD holders in these fields.

Although each field in aggregate behaves in similar ways, we see differences across the fields when we disaggregate results by gender (Fig 1). In the biological sciences, there has been a persistent gender gap in no job offers since 2000, with about 5 percent more women indicating they have no offers at the end of their programs relative to men. In engineering, the gender gap on this measure closed around 1990 but has been widening since then—in 2010, the gender gap was at its largest at around 7.5 percent. The physical sciences show a different pattern, as the gender gap in no offers has closed in recent years.

We also identify key patterns for race (Fig 2). For biological sciences, there is a persistent gap in no offers (approximately 5 percent) between White PhD recipients and Asian, Black and Hispanic recipients. Since the mid-2000’s, Black and Hispanic PhD recipients in the biological sciences have trended toward the Asian PhD recipients in having a higher percentage of PhD recipients finish their programs with no offers relative to White PhD recipients. The gap between White PhD recipients and the other race categories is widest for engineering, with Hispanic and Black PhD recipients eclipsing Asian PhD recipients with no job offers in the most recent years. And much like the finding for gender in physical sciences, the gap between White students and the other racial groups has been narrowing in the physical sciences in recent years, with a high amount of fluctuation for Black students because of low overall numbers of doctoral recipients.

We extended our analyses of differences across gender and race by accounting for other key variables that have been connected to career choices for doctoral students in prior literature, namely family variables (i.e., marital status, dependents) and doctoral funding mechanism. We considered models with main effects as well as interaction effects between the gender and race variables and the family and funding variables.

### Differences by gender

Across all three field categories, female respondents reported having no job offers at a higher rate than male respondents, with all differences statistically significant with the inclusion of other main effect and control variables (see Table 4). As seen in Table 4, females in biology and engineering reported a higher difference, with women 25 percent more likely to have no prospects in biology and 29 percent more likely in engineering. Women in the physical sciences had a more modest, but still statistically significant, difference at 9 percent. It is important to note that we also found several statistically significant interaction effects with our other variables of interest, particularly marital status. We discuss these findings in the relevant subsequent sections, which explain differences by gender.

### Differences by race

Of the nine racial main effect comparisons (Asian v. White, Black v. White, and Hispanic v. White for each of three fields), each non-White racial group had significantly higher percentages of no job offers in comparison to White respondents. Across all three fields, Asian respondents had the highest odds ratios, all at or above 40 percent more likely to have no job offers; Hispanic respondents had the lowest odds ratios of the non-White racial groups, reaching as high as 30 percent more likely than White respondents to have no job offers. Asian engineers and physical scientists and Black engineers had some of the highest odds ratios in the models, at 64 percent and 61 percent more likely to have no offers, respectively.

In biology, gender-race interaction effects were statistically significant for all three interactions (i.e., Female-Asian, Female-Black, and Female-Hispanic). This relationship is depicted in Fig 3A. Whereas female respondents report a higher percentage of no job offers than males across all racial groups, the gender gap is narrowest for White PhDs. In engineering, only the Female-Black interaction was significant with a negative coefficient. None of the gender-race interactions were statistically significant in physical sciences.

### Relationships with family variables

We found similar patterns across all three fields for the relationship between family variables and no job prospects. Being married and having more dependent children was associated with a reduced likelihood that the respondent had no job prospects.

**Fig 4:**
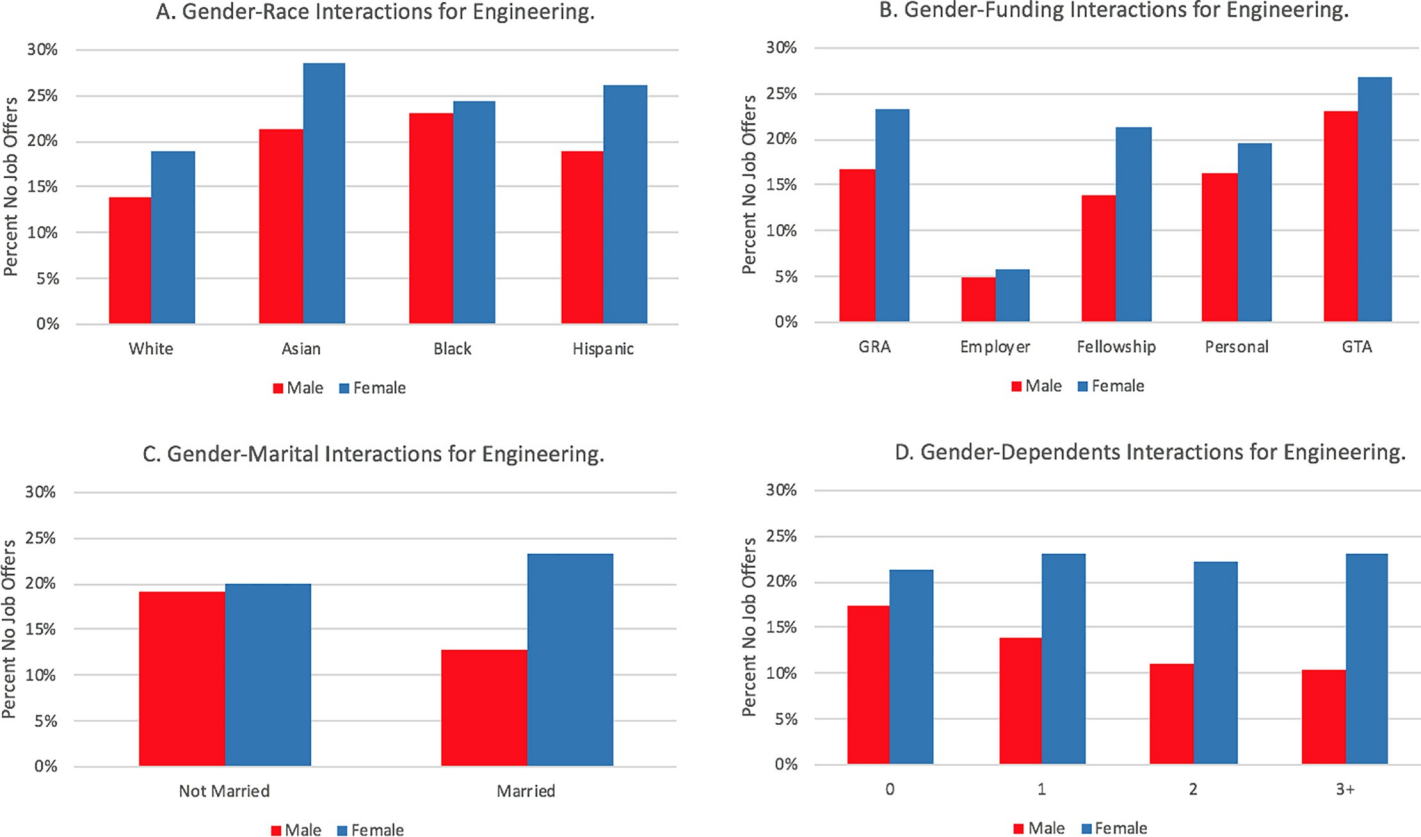
Interaction plots of significant gender interactions for engineering.

**Fig 5:**
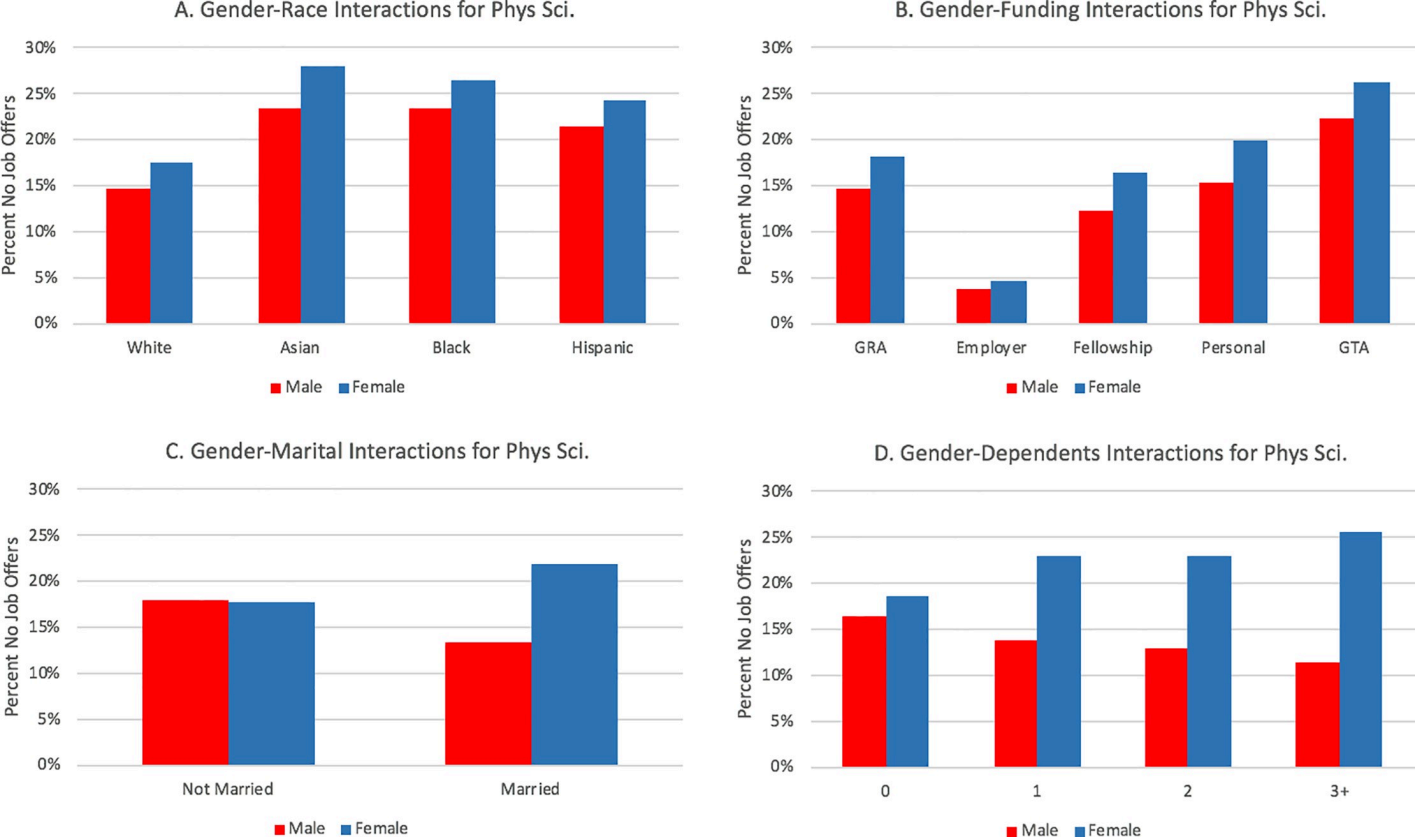
Interaction plots of significant gender interactions for physical sciences.

In addition to the strong main effect relationships, respondents in each field category demonstrated a strong interaction effect with gender (see Tables B1, B2, and B3 in S2 Appendix). The interaction plots, shown for each field in Fig 3C, Fig 4C and Fig 5C, suggest gender effects are driven primarily by a lower rate of no job offers among married men, particularly in biology and engineering. Both unmarried men and women have similar percentages of no job offers, and unmarried women in physical sciences had slightly lower percentages of no job offers than men.

Similar interaction effects appear for dependent children. Within biology (Fig 3D) and the physical sciences (Fig 5D), females show an increasing prevalence of no job offers as their number of dependents increase; the opposite relationship is apparent for males in those fields. Within engineering, males also have a negative slope, whereas females have a flat relationship (i.e., there are no noticeable differences for females who have zero, one, two, or three or more dependents in terms of not having a job offer).

**Fig 6:**
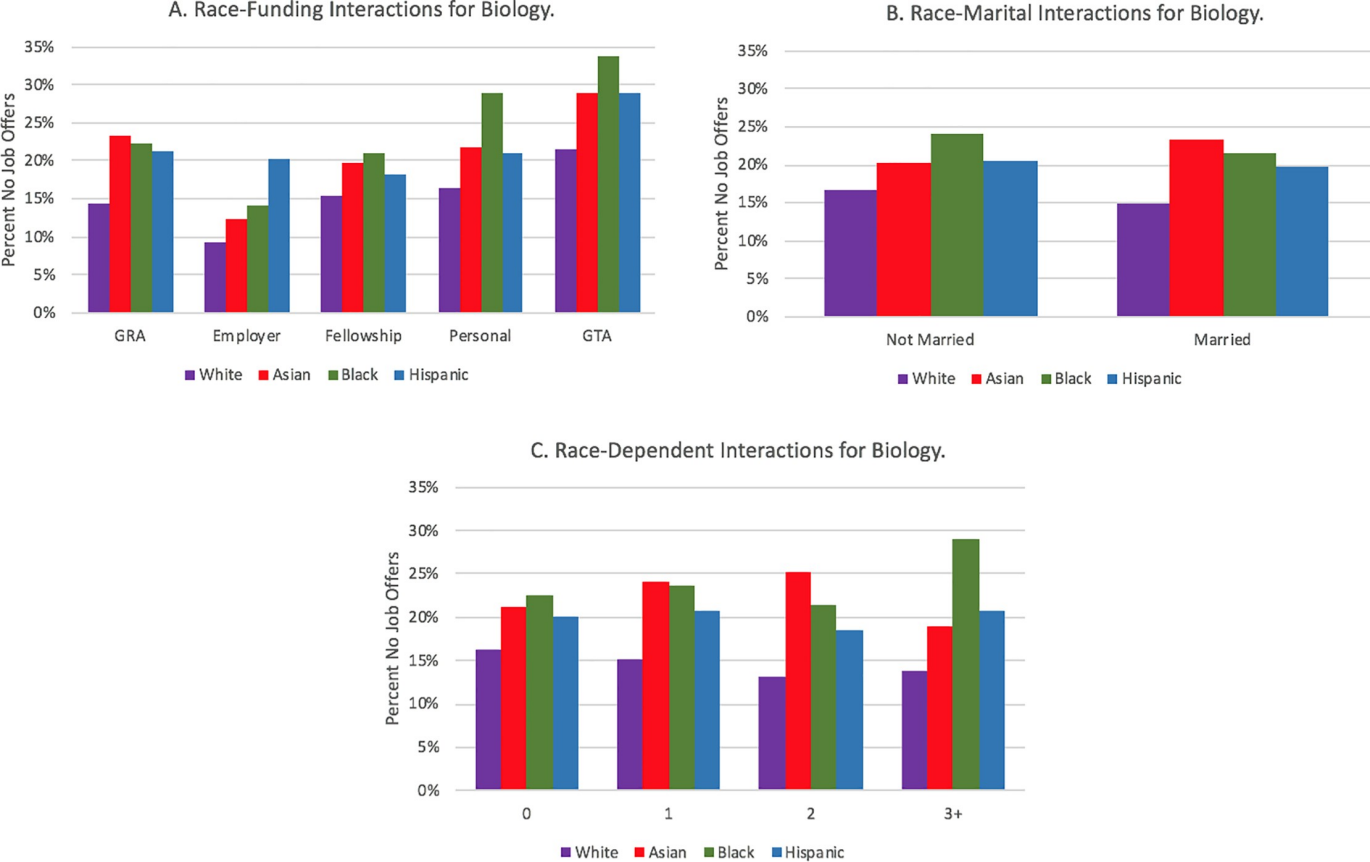
Interaction plots of significant race interactions for biological sciences.

**Fig 7:**
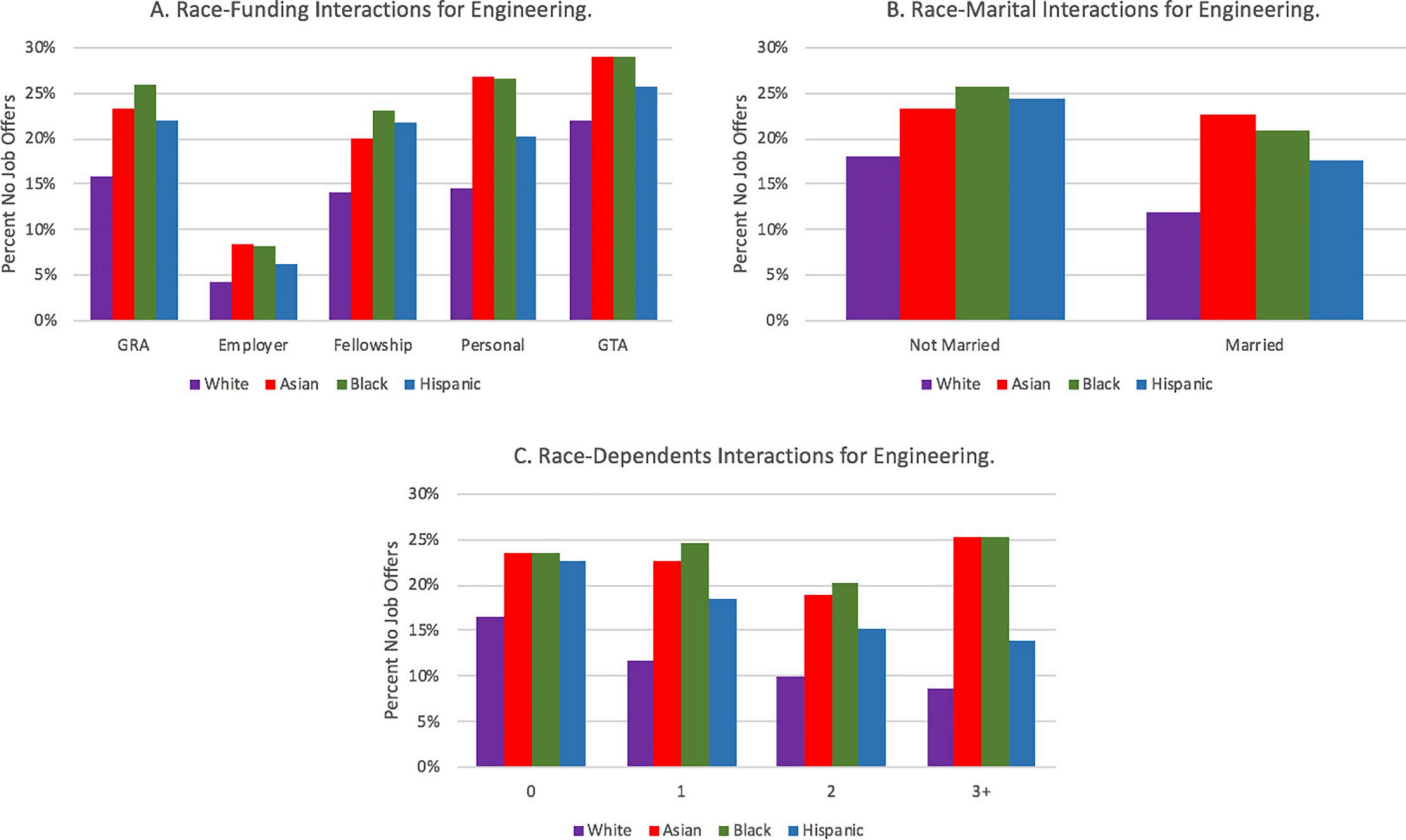
Interaction plots of significant race interactions for engineering.

There were also multiple statistically significant interaction effects by race. In biology (Fig 6B), a greater percentage of unmarried Black PhD recipients have no job offers. Also, as the number of dependents increases (Fig 6C), a smaller percentage of White PhD biology recipients have no offers, whereas the other race categories fluctuate highly. Among engineering doctoral recipients with dependents, the racial gap in no job offers tends to widen between White students and Hispanic students and Black students (Fig 7B). It should be noted, however, that these results may be drive through low numbers of respondents in these groups. There were no statistically significant race interactions for either the married or dependents variable for the physical sciences.

### Relationships with graduate funding

Some of the largest coefficients in the model were for primary funding mechanisms. Within biology, relative to students who were primarily funded on a GRA, students funded on a GTA were 48 percent more likely to have no job offers, and students who funded their own education were 11 percent more likely to have no job offers. Students funded by an employer were less likely than GRAs to have no job offers. Similar patterns emerged in engineering and physical sciences for the relationship between the employer, fellowship, and teaching assistant funding mechanisms. Engineering and physical science respondents with personal funding had a lower likelihood of no job offers, which was the opposite relationship than respondents in biology.

**Fig 8:**
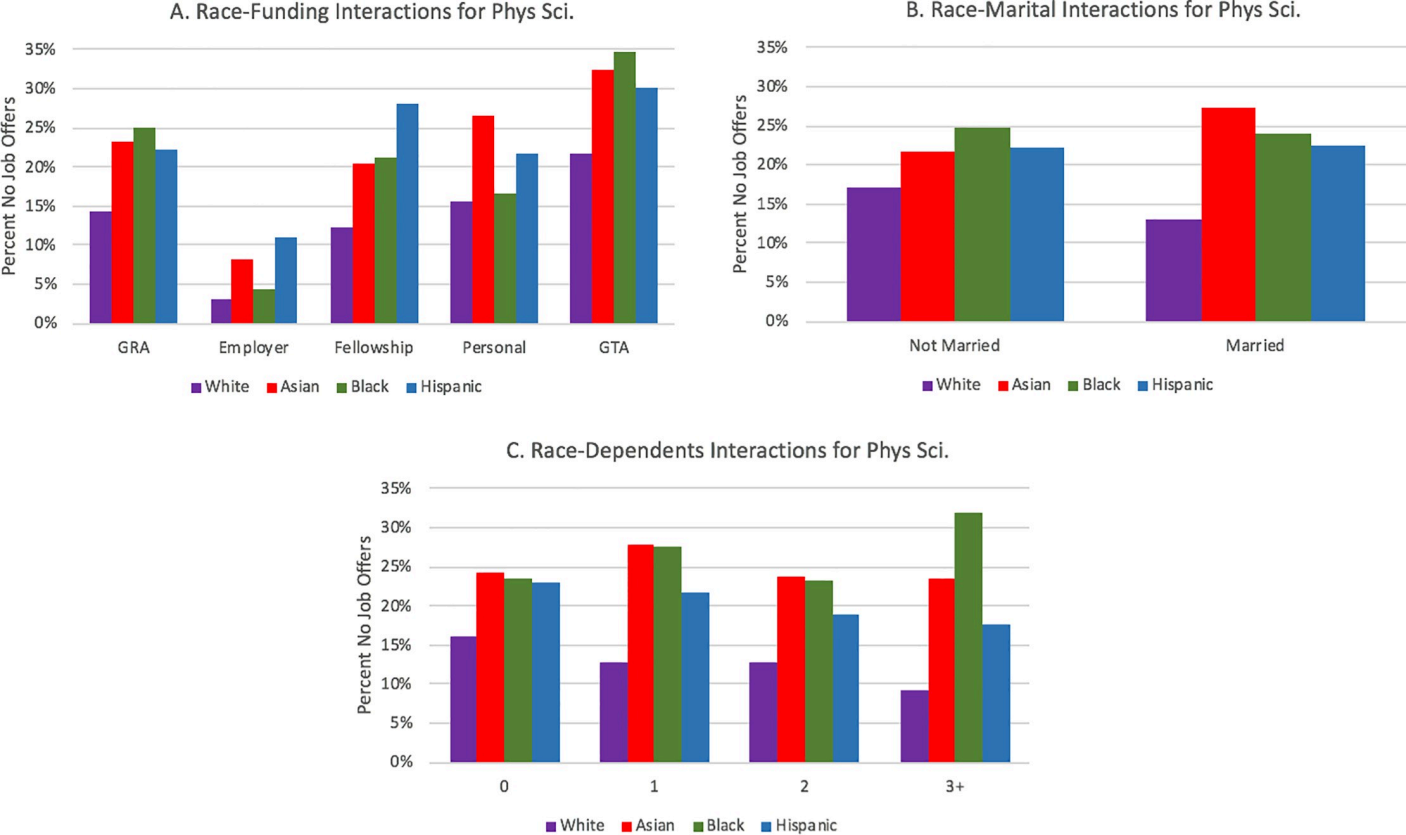
Interaction plots of significant race interactions for physical sciences.

Each field had at least one statistically significant gender and race interaction effect within each funding type. In biology (Fig 3B), the gender gap was wider for students funded via fellowships or personal funding relative to the other funding mechanisms. For interactions by race/ethnicity, Hispanic students with employer funding and Black students on fellowships or who were self-funded displayed distinctively higher rates of no offers. Like the biological sciences, the gender gap in no offers is most pronounced in engineering for women who are funded via fellowships; it is least pronounced for students funded via teaching assistantships or employer-based funding (see Fig 4B). The largest racial gaps in no job offers for physical sciences doctoral recipients by funding mechanism are between Black and White students funded via GRAs and fellowships (Fig 8A). Black and White GTAs, however, are roughly the same with respect to no job offers, whereas Asian GTAs in particular have a higher percentage of no offers.

Finally, like the other fields, students in physical sciences funded on a GTA were 42 percent more likely to have no job offers relative to students funded on a GRA, and employer-funded students and fellowship recipients were less likely to have no offers than students funded by a GRA. There was not a significant difference in no job offers across funding mechanisms by gender, but we observed interaction effects for Black students funded via fellowships, TAs, and personal finances.

### Robustness checks

Our first robustness check comparing respondent data since 2001 (i.e., when the outcome variable survey item was reworded) to the full time period of interest (since 1976) yielded only two changes to the main effect variables across the three field-level analyses. A comparison of the main effects model and the model filtered for graduation years 2001 to 2016 (Table B4 in S2 Appendix) within the biological sciences identifies one significant coefficient sign change (i.e., personal funding) and a newly significant main effect for Hispanic doctoral recipients. All other main effect variables within the three fields exhibited no changes to direction or significance. Because these minimal shifts were limited to a variable with a smaller sample size, we do not believe the wording change affected the initial model results based on the full period of record.

The second robustness check compared the main effect coefficients and odds ratios for each field in Table 4 to the field-level interaction effects of the main effect variables found in Table B5 in S2 Appendix. Eight of ten of the possible engineering to biological sciences interactions and six of ten physical sciences to biological sciences interactions were statistically significant. In comparison to the odds ratios in Table 4, all statistically significant interactions correspond with a notable difference in the odds ratio. For example, for female respondents, the statistically significant difference between physical sciences and biological sciences sits in parallel to an odds ratio difference of 1.09 to 1.25. In contrast, the non-significant difference between engineering and biological sciences relate to an odds ratio comparison of 1.29 to 1.25. These and similar results for other main effect variables provide evidence to support our main effect comparisons of separate regression models by field.

Finally, our robustness check using SDR data yielded findings about the persistent difficulty of finding employment beyond graduation. As shown in Table B6 in S2 Appendix, across all fields, a higher percentage of SED respondents who were seeking work but had no job offers at the time of survey completion (i.e., at the end of their graduate programs) were still not working within three years of graduation (8.9% compared to 4.1% of respondents across all fields who had a job or were not seeking a job at graduation, see Table B6 in S2 Appendix). This difference was consistent across all three fields. When we subset the data to include only doctoral recipients from the fiscal years 2001 through 2016, the differences become more stark, with the gap widening for this most recent time window.

We also utilized SDR data to gain further insight into factors relating to employment patterns, in particular differences by gender and marital status. Focusing on SED respondents who initially reported that they were seeking work but had no job offers and were working in a job unrelated to their doctoral degree within three years of the degree, we identified two factors with notable differentiation (see Table B7 in S2 Appendix). Almost three-fourths (72 percent) of married females reported job location as a factor in working in unrelated employment to their degree. This compares to 51 percent for married males, 53 percent for unmarried females, and 47 percent for unmarried males. Family demands also had a disproportionate response from married respondents, with stark differences between males (30 percent) and females (51 percent). Unmarried males and females were more similar at 15 percent and 12 percent, respectively.

## Discussion

This research extends prior lines of inquiry into the career outcomes of doctoral recipients by examining the differential prevalence of no job offers at the time of degree by gender and race, while accounting for the influence of family and funding variables. Observed trends over the past 15 years align with current concerns of an oversaturated labor market [5]. Across all three fields examined, the prevalence of no job offers increased over time, reaching all-time highs in the most recent years. Much of the concern regarding the occupational outlook for recent doctoral recipients has focused on the stagnant or shrinking labor market for tenure-track faculty members [12], which improved advising practices for non-academic careers are designed to address [2]. Our analysis expands the universe to job offers in all job sectors. Whether our results are also reflective of a tightening academic and non-academic labor market or larger systemic issues in doctoral education in general deserves closer examination.

Our findings lend credence to the growing base of literature that demonstrates that women and underrepresented minority PhDs have disparate outcomes when navigating the post-PhD labor market. Of particular concern is the finding that among fellowship students, there are gender and race gaps in job offers. Given the limitations of our data, our hope is that follow-up research seeks to illuminate further the underlying systemic mechanisms that influence these inequities. One of the most promising areas for deeper exploration with recent increased interest is that of hiring practices, both for women (e.g., [14]) and URMs (e.g., [15]). Despite increased calls for the hiring of underrepresented populations in STEM fields, our findings demonstrate that women and URM doctoral recipients experience higher rates of no job offers at the time of graduation than male and white SED respondents. Importantly, however, we found that this gender gap appears to relate strongly to family variables (i.e., marital status and dependents) and funding mechanism, and additional research is needed to interrogate those relationships more directly.

Our findings of significant relationships with family factors illuminate a potentially rich area for further exploration and offer some ideas for practical implications. Specifically, the uniquely positive interaction of marriage and dependent children for men deserves additional follow-up. Our results align with prior research that show a gendered relationship for family and career variables (e.g., [32]); however, many of these studies are framed as a negative influence for women. Our findings that being married or having one or more dependents reduces the odds of male respondents having no job offers align with prior research on supply-side dynamics (e.g., [36]). That research posits that in heterosexual couples, male careers typically take precedence over the careers of female spouses or males feel more responsibility to obtain a job to support their family. These prior patterns are further bolstered by our SDR analyses which showed that married females take employment in jobs unrelated to their doctoral degrees because of familial demands and job location more frequently than married males. Although we saw this similar phenomenon for doctoral recipients, a causal explanation requires additional research.

It is important to note that our data do not include information about the type of job that respondents with no initial job offers in this study eventually take. One potential explanation is that some respondents were waiting for a dream job, such as a tenure-track faculty position that may hire on a fixed annual hiring cycle. It is not surprising that unmarried PhDs and those without dependents have higher rates of no job offers, as they may have fewer financial pressures and can take more time to search for a particularly desirable position. However, it is more difficult to interpret the gender differences for respondents who have spouses or dependents. One interpretation is that married women may have more flexibility to search for a job with particularly good fit because their partner can provide financial support. On the other hand, this financial and timing flexibility may come at the cost of geographic flexibility if married women are more constrained to find a position in the same city where their partner is employed. We know from prior research, for example, that marriage increases women’s likelihood of part-time, non-tenure-track employment [34, 35], which would be more likely if they are constrained to search for academic jobs in a specific location. Our research identifies a broad systematic difference that requires more targeted analyses to understand the rationale underlying job search strategies of PhDs.

In addition to exploring demand-side inequities, our findings can also influence, and perhaps could subsequently be influenced by, further examination of the supply-side aspects of how doctoral students conduct their job searches generally. Prior research has demonstrated that women [41] and URM [31] doctoral students have expressed desires for careers in varied sectors relative to White males. In addition, Ceci et al. [23] identified that such crucial variables as work-life balance and family friendly work environments can influence women’s decisions to “opt out” or “lean in” to certain careers. For example, Denton et al. [44] used SDR data to find that five or six years after PhD completion, Hispanic, Black and female engineers are more likely to be working in education sectors, whereas Asian, White and male engineers are more likely to be working in industry. In an interview study of Black female and Latina engineering faculty, DeCuir-Gunby et al. [45] found that several chose academia because of challenges experienced while working in industry. Further research can explore how doctoral earners’ career intentions may influence their job search and ultimate initial employment.

The strong and consistent relationship of primary funding mechanism is also owed further examination. Our findings showed that doctoral recipients funded via a research assistantship had lower percentages of no offers than graduates funded with a teaching assistantship. This result aligns with previous studies showing more positive outcomes for students funded via research assistantships (e.g., [20]). We wonder whether: 1) GTAs are awarded systematically to students who might not be as competitive academically upon matriculation, 2) the nature of the GTA experience, such as focusing on tasks that may not spur certain kinds of research development as a doctoral student, might lead to differentiation in career outcomes, or 3) a combination of these explains the discrepancy. Regardless of the underlying mechanisms, our findings suggest that programs should pay increased attention to the implications of its allocation of funding mechanisms across students.

Unlike some prior work, however, we find a positive relationship for career outcomes (i.e., lower chances of no offers) across all three fields for fellowships in comparison to research assistantships. One caveat to consider in interpreting our results is that we included no controls for students’ prior academic ability because of data limitations. Similar to the allocation of GTAs, perhaps these findings are driven through the awarding of fellowships to students who may be most academically competitive upon their admission to the doctoral program, which could relate to differences in finding post-PhD employment.

More striking, however, was the result of the interaction effect between gender and fellowships—relative to males, a larger percentage of females in the biological sciences and engineering who were funded via fellowships did not have job offers. The interaction effects for other subpopulations were also negative, as more Asian, Black, and Hispanic graduates in biology funded on fellowships had no job offers than White graduates. As fellowships are often used for the recruitment of underrepresented populations, an increased understanding of the disparate relationship of fellowships with career outcomes is critical. If students recruited through fellowship opportunities have weakened employment outcomes as compared to students recruited and supported via other mechanisms, strategies seeking to enhance diversity in STEM doctoral education may be misguided. Further, as funding mechanisms are one aspect of doctoral education over which academic departments have some kind of programmatic control, obtaining greater clarity on the relationship between these funding mechanisms and subsequent career outcomes would hopefully lead to more purposeful practices within doctoral education.

Finally, we conclude with a relevant discussion on the crucial role of disciplinary field. Foundational work in doctoral education (e.g., [46]) has demonstrated the role of the department and, by extension, the discipline or field in the organization of doctoral education. In acknowledgment of these differences, we chose to focus our attention of this phenomenon on each field separately. Comparisons of the relationships at face value illuminate curious differences that would serve as a useful basis for further research. Further investigation could provide insight into “best practices” currently used in fields that do not show gaps in job offers across the demographic, family, and funding mechanism variables investigated in this study. Additional work can begin to parse our within discipline differences by exploring differences by subfield, e.g. chemistry versus physics.

## Supporting information

S1 Appendix Sub field summaries. (DOCX)

S2 Appendix Logistic regression tables. (DOCX)

## References

[1] Conti A, Visentin F. Science and engineering Ph.D. students’ career outcomes, by gender. PLoS ONE. 2015;10(8):e0133177 10.1371/journal.pone.0133177 26244797 PMC4526637

[2] St. Clair R, Hutto T, MacBeth C, Newstetter W, McCarty NA, Melkers J. The “new normal”: Adapting doctoral trainee career preparation for broad career paths in science. PLos ONE. 2017;1–19. 10.1371/journal.pone.0177035 PMC5443479 28542304

[3] Austin AE, McDaniels M. (2006). Preparing the professoriate of the future: Graduate student socialization for faculty roles. High Educ: Handb of Theory and Res. 2016;21:397–456.

[4] Allum JR, Kent JD, McCarthy MT. Understanding PhD Career Pathways for Program Improvement. Washington (DC): Council of Graduate Schools 2014.

[5] Cyranoski D, Gilbert N, Ledford H, Nayar A, Yahia M. The PhD Factory. Nature, 2011;472:276–279. 10.1038/472276a 21512548

[6] Turk-Bicacki L, Berger A, Haxton C. The Nonacademic Careers of STEM PhD Holders. Washington (DC): American Institutes of Research 2014.

[7] Roach M, Sauermann H. A taste for science? PhD scientists’ academic orientation and self-selection into research careers in industry. Res Policy. 2014;9(2010):422–434.

[8] Thiry H, Laursen SL, Loshbaugh HG. “How do I get from here to there?”: A examination of Ph.D. science students’ career preparation and decision making. Int J Doctoral Stud, 2015;10:237–256.

[9] June AW. Why college still scarely track Ph.D.s. The Chron of High Educ. 2016 8—[cited 2019 Jun 3]. Available from https://www.chronicle.com/article/Why-Colleges-Still-Scarcely/237412

[10] Council of Graduate Schools. Graduate education leaders issue global statement on career outcomes for students. [cited 2019 Jun 3]. Available from http://cgsnet.org/graduate-education-leaders-issue-global-statement-career-outcomes-students.

[11] Blank R, Daniels RJ, Gilliland G, Gutmann A, Hawgood S, Hrabowski FA, et al A new data effort to inform career choice in biomedicine. Science. 2017;358(6369):1388–1389. 10.1126/science.aar4638 29242335

[12] Gould J. How to build a better PhD. Nature. 2015;528:22–25. 10.1038/528022a 26632571

[13] National Center for Science and Engineering Statistics. (2017). Women, Minorities, and Persons with Disabilities in Science and Engineering. Washington (DC): National Science Foundation 2017.

[14] Glass C, Minnotte KL. Recruiting and hiring women in STEM fields. J Divers High Educ. 2010;3(4):218–229.

[15] Gibbs Jr KD, Basson J, Xierali IM, Broniatowski DA. Decoupling of the minority PhD talent pool and assistant professor hiring in medical school basic science departments in the U.S. eLife. 2016;2016(5):e21393. 10.7554/eLife.21393 PMC5153246 27852433

[16] Gumpertz M, Durodoye R, Griffith E, Wilson A. Retention and promotion of women and underrepresented minority faculty in science and engineering at four large land grant institutions. PLoS ONE. 2017;12(11):e0187285 10.1371/journal.pone.0187285 29091958 PMC5665535

[17] Bureau of Labor Statistics. Unemployment rates and earnings by educational attainment, 2017. [cited 2019 Jun 3]. Avaialble from https://www.bls.gov/emp/ep_chart_001.htm.

[18] National Science Foundation. 2017 Doctorate Recipients from U.S. Universities. Washington (DC): National Science Foundation 2018.

[19] U.S. Census Bureau. Educational Attainment in the United States: 2018. [cited 2019 Jun 3]. Available from https://www.census.gov/data/tables/2018/demo/education-attainment/cps-detailed-tables.html

[20] Ehrenberg RG, Mavros P. Do doctoral students‘ financial support patterns affect their times-to-degree and completion probabilities? Natl Bur of Econ Rese. 1992;No. w4070.

[21] Millett CM, Nettles MT. Expanding and cultivating the Hispanic STEM doctoral workforce: Research on doctoral student experiences. J Hispanic High Educ. 2006;5(3):258–287.

[22] Blume-Kohout M, Adhikari D. Training the scientific workforce: Does funding mechanism matter? Res Policy, 2016;45:1291–1303. 10.1016/j.respol.2016.03.011 28461709 PMC5409136

[23] Ceci SJ, Ginther DK, Kahn S, Williams WM. Women in academic science: A changing landscape. Psychol Sci Public Interest. 2014;15(3):75–141. 10.1177/1529100614541236 26172066

[24] Blickenstaff JC. Women and science careers: Leaky pipeline or gender filter? Gender Educ, 2005;17:369–386.

[25] Hill C, Corbett C, St. Rose A. Why so few? Women in science, technology, engineering, and mathematics. Washington (DC): American Association of University Women 2010.

[26] Moss-Racusin CA, van der Toorn J, Dovidio JF, Brescoll VL, Graham MJ, Handelsman J. Scientific diversity interventions. Science. 2014;343:615–616. 10.1126/science.1245936 24503840

[27] Williams WM, Ceci SJ. (2015). National hiring experiments reveal 2:1 faculty preference for women on STEM tenure track. Proceedings of the Natl Acad Sci. 2015;112(17):5360–5365. 10.1073/pnas.1418878112 PMC4418903 25870272

[28] Garrison H. Underrepresentation by race-ethnicity across stages of U.S. science and engineering education. CBE-Life Sci Educ. 2013;12:357–363. 10.1187/cbe.12-12-0207 24006384 PMC3763003

[29] Jarvis ED. Surviving as an underrepresented minority scientist in a majority environment. Mol Biol Cell, 2015;26:3692–3696. 10.1091/mbc.E15-06-0451 26515973 PMC4626054

[30] Gibbs Jr. KD, Griffin KA. What do I want to be with my PhD? The roles of personal values and structural dynamics in shaping the career interests of recent biomedical science PhD graduates. CBE-Life Sci Educ. 2013;12:711–723. 10.1187/cbe.13-02-0021 24297297 PMC3846521

[31] Gibbs Jr. KD, McGready J, Bennett JC, Griffin K. Biomedical science Ph.D. career interest patterns by race/ethnicity and gender. PLoS ONE, 2014;9(12):1–18. 10.1371/journal.pone.0114736 PMC4262437 25493425

[32] Morrison E, Rudd E, Nerad M. Onto, up, off the academic faculty ladder: The gendered effects of family on career transitions for a cohort of social science Ph.D.s. The Rev High Educ. 2011;34(4):525–553.

[33] De Welde K, Laursen SL. The glass obstacle course: Informal and formal barriers for women Ph.D. students in STEM fields. Intl J Gender Sci Tech. 2011;3(3):572–595.

[34] Perna LW. The relationship between family responsibilities and employment status among college and university faculty. J High Educ. 2001;72(5):584–611.

[35] Long JS. From Scarcity to Visibility: Gender differences in the careers of doctoral scientists and engineers. Washington (DC): National Academy Press 2001.

[36] Harper EP, Baldwin RG, Gansneder BG, Chronister JL. Full-time women faculty off the tenure track: Profile and practices. Rev High Educ. 2001;24(3):237–57.

[37] Worthen BR, Gardner MK. A second look at the relation of research assistantships and research productivity. Annual Meeting of the American Educational Research Association. 1988.

[38] Buchmueller TC, Dominitz J, Hansen WL. Graduate training and the early career productivity of Ph.D. economists. Econ Educ Rev. 1999;18:65–77.

[39] Mendoza P, Villarreal P III, Gudnerson A. Within-year retention among Ph.D. students: The effect of debt, assistantships, and fellowships. Res High Educ. 2014;55:650–685.

[40] National Science Foundation. Survey of Earned Doctorates. [cited 2019 Jun 3]. Available from nsf.gov

[41] Sauermann H, Roach M. Science PhD Career Preferences: Levels, Changes, and Advisor Encouragement. PLoS ONE. 2012;7(5):e36307 10.1371/journal.pone.0036307 22567149 PMC3342243

[42] Mervis J. ‘Employment crisis’ for new Ph.D.s is an illusion. Science. 2016 - [cited 2019 Jun 3]. Available from http://www.sciencemag.org/careers/2016/05/employment-crisis-new-phds-illusion. 10.1126/science.352.6288.880 27199396

[43] Byars-Winston A, Estrada Y, Howard C. Increasing STEM retention for underrepresented students: Factors that matter. Madison (WI): Center for Education and Work 2008.

[44] Denton M, Choe NH, Nguyen KA, Borrego MJ, Knight DB, Bortz WW, et al Predictors of Engineering Doctoral Students’ Future Career Sector. Proceedings of Annual Meeting of the American Society of Engineering Education. 2019.

[45] DeCuir-Gunby JT, Grant C, Gregory BB. (2013). Exploring career trajectories for women of color in engineering: The experiences of African American and Latina engineering professors. J of Women and Minorities in Sci and Eng. 2013;19(3),209–225.

[46] Golde CM. The role of the department of discipline in doctoral student attrition: Lessons from four departments. J High Educ. 2005;76(6):669–700.

