# Supplementary material for: Illuminating systematic differences in no job offers for STEM doctoral recipients
Source: PLoS One. 2020 Apr 29;15(4):e0231567. doi: 10.1371/journal.pone.0231567 (PMC7190089; doi:10.1371/journal.pone.0231567)
Supplement: S1 Appendix — (DOCX) [file pone.0231567.s001.docx]

**APPENDIX A: Sub Field Summaries**

| **Table A1. Field of Study Breakdown by Biological Sciences, Engineering, and Physical Sciences as of the 2016 SED** | | | |
| --- | --- | --- | --- |
| ***BIOLOGICAL/BIOMEDICAL SCIENCES*** | | | |
| Anatomy | Endocrinology | | Pathology, Human & Animal |
| Bacteriology | Entomology | | Pharmacology, Human & Animal |
| Biochemistry | Environmental Toxicology | | Physiology, Human & Animal |
| Bioinformatics | Epidemiology | | Plant Genetics |
| Biomedical Sciences | Evolutionary Biology | | Plant Pathology/Phytopathology |
| Biometrics & Biostatistics | Genetics/Genomics, Human & Animal | | Plant Physiology |
| Biophysics | Immunology | | Structural Biology |
| Biotechnology | Marine Biology & Biological Oceanography | | Toxicology |
| Botany/Plant Biology | Microbiology | | Virology |
| Cancer Biology | Molecular Biology | | Wildlife Biology |
| Cell Biology & Histology | Molecular Medicine | | Zoology |
| Computational Biology | Neurosciences & Neurobiology | | Biology/Biomedical Sciences, General |
| Developmental Biology/Embryology | Parasitology | | Biology/Biomedical Sciences, Other |
| Ecology |  | |  |
| ***ENGINEERING*** | | | |
| Aerospace, Aeronautical, & Astronautical Engineering | | Materials Science Engineering | |
| Agricultural Engineering | | Mechanical Engineering | |
| Bioengineering & Biomedical Engineering | | Metallurgical Engineering | |
| Chemical Engineering | | Nuclear Engineering | |
| Civil Engineering | | Ocean Engineering | |
| Communications Engineering | | Operations Research | |
| Computer Engineering | | Petroleum Engineering | |
| Computer Science | | Polymer & Plastics Engineering | |
| Electrical, Electronics, & Communications Engineering | | Robotics | |
| Engineering Management & Administration | | Structural Engineering | |
| Engineering Mechanics | | Systems Engineering | |
| Engineering Physics | | Transportation & Highway Engineering | |
| Engineering Science | | Engineering, General | |
| Environmental/Environmental Health Engineering | | Engineering, Other | |
| Geotechnical & Geoenvironmental Engineering | | Computer & Information Systems, General | |
| Industrial & Manufacturing Engineering | | Computer & Information Systems, Other | |
| Information Science & Systems | |  | |
| ***PHYSICAL SCIENCES*** | | | |
| Astronomy | Astrophysics | | Astronomy, Other |
| Atmospheric Chemistry & Climatology | Meteorology | | Atmospheric Science/Meteorology, Other |
| Atmospheric Physics & Dynamics | Atmospheric Science/Meteorology, General | |  |
| Analytical Chemistry | Organic Chemistry | | Theoretical Chemistry |
| Chemical Biology | Physical Chemistry | | Chemistry, General |
| Inorganic Chemistry | Polymer Chemistry | | Chemistry, Other |
| Medicinal Chemistry |  | |  |
| Geochemistry | Geophysics & Seismology | | Stratigraphy & Sedimentation |
| Geology | Mineralogy & Petrology | | Geological & Earth Sciences, General |
| Geomorphology & Glacial Geology | Paleontology | | Geological & Earth Sciences, Other |
| Hydrology & Water Resources | Marine Sciences | | Oceanography, Chemical & Physical |
| Ocean/Marine, Other |  | |  |
| Acoustics | Medical Physics/Radiological Science | | Plasma/Fusion Physics |
| Applied Physics | Nuclear Physics | | Polymer Physics |
| Atomic/Molecular/Chemical Physics | Optics/Photonics | | Physics, General |
| Biophysics | Particle Physics | | Physics, Other |
| Condensed Matter/Low Temperature Physics |  | |  |
