# Supplementary material for: Illuminating systematic differences in no job offers for STEM doctoral recipients
Source: PLoS One. 2020 Apr 29;15(4):e0231567. doi: 10.1371/journal.pone.0231567 (PMC7190089; doi:10.1371/journal.pone.0231567)
Supplement: S2 Appendix — (DOCX) [file pone.0231567.s002.docx]

**APPENDIX B: Logistic Regression Tables**

| **Table B1. Model with Interaction Effects for Biological Sciences.** | | | | | | |
| --- | --- | --- | --- | --- | --- | --- |
|  | | Main Effects | Gender Interactions | Race Interactions | | |
|  |  |  | Female | Asian | Black | Hispanic |
| Intercept | | -3.085 (0.147) *** |  |  |  |  |
| GENDER | |  |  |  |  |  |
|  | Female | 0.841 (0.131) *** |  |  |  |  |
| RACE | |  |  |  |  |  |
|  | Asian | -0.127 (0.284) | 0.223 (0.059) *** |  |  |  |
|  | Black | 0.368 (0.376) | 0.245 (0.099) * |  |  |  |
|  | Hispanic | 0.446 (0.341) | 0.190 (0.078) * |  |  |  |
| FUNDING | |  |  |  |  |  |
|  | Employer | -0.920 (0.130) *** | 0.124 (0.173) | -0.064 (0.273) | -0.096 (0.472) | 0.729 (0.343) * |
|  | Fellowship | -0.114 (0.030) *** | 0.145 (0.039) *** | -0.215 (0.065) *** | -0.111 (0.116) | -0.252 (0.090) ** |
|  | Personal | 0.208 (0.040) *** | -0.015 (0.056) | -0.232 (0.121) | -0.057 (0.181) | -0.379 (0.156) * |
|  | GTA | 0.509 (0.034) *** | -0.112 (0.049) * | -0.305 (0.108) ** | -0.083 (0.178) | -0.196 (0.143) |
| FAMILY | |  |  |  |  |  |
|  | Married | -0.303 (0.025) *** | 0.380 (0.033) *** | -0.030 (0.063) | -0.210 (0.101) * | -0.027 (0.079) |
|  | Dependents | -0.152 (0.015) *** | 0.069 (0.022) ** | 0.069 (0.047) | 0.109 (0.055) * | 0.136 (0.051) ** |
| * p<0.1; ** p<0.01; p<0.001 | | | | | | |

| **Table B2. Model with Interaction Effects for Engineering.** | | | | | | |
| --- | --- | --- | --- | --- | --- | --- |
|  | | Main Effects | Gender Interactions | Race Interactions | | |
|  |  |  | Female | Asian | Black | Hispanic |
| Intercept | | -2.421 (0.254) *** |  |  |  |  |
| GENDER | |  |  |  |  |  |
|  | Female | 0.327 (0.187) |  |  |  |  |
| RACE | |  |  |  |  |  |
|  | Asian | 0.012 (0.252) | 0.126 (0.070) |  |  |  |
|  | Black | -0.048 (0.414) | -0.087 (0.117) |  |  |  |
|  | Hispanic | 0.512 (0.381) | 0.020 (0.110) |  |  |  |
| FUNDING | |  |  |  |  |  |
|  | Employer | -1.490 (0.081) *** | -0.147 (0.185) | -0.038 (0.201) | -0.090 (0.308) | 0.359 (0.303) |
|  | Fellowship | -0.253 (0.032) *** | 0.183 (0.056) ** | -0.011 (0.074) | -0.097 (0.133) | 0.181 (0.112) |
|  | Personal | -0.098 (0.040) * | -0.209 (0.086) * | 0.204 (0.099) * | -0.168 (0.192) | 0.177 (0.176) |
|  | GTA | 0.346 (0.043) *** | -0.261 (0.097) ** | -0.095 (0.112) | -0.500 (0.260) | -0.099 (0.202) |
| FAMILY | |  |  |  |  |  |
|  | Married | -0.440 (0.026) *** | 0.445 (0.050) *** | 0.043 (0.067) | -0.153 (0.123) | -0.036 (0.104) |
|  | Dependents | -0.202 (0.015) *** | 0.165 (0.032) *** | 0.049 (0.042) | 0.178 (0.058) ** | 0.006 (0.064) |
| * p<0.1; ** p<0.01; p<0.001 | | | | | | |

| **Table B3. Model with Interaction Effects for Physical Sciences.** | | | | | | |
| --- | --- | --- | --- | --- | --- | --- |
|  | | Main Effects | Gender Interactions | Race Interactions | | |
|  |  |  | Female | Asian | Black | Hispanic |
| Intercept | | -8.888 (43.95) |  |  |  |  |
| GENDER | |  |  |  |  |  |
|  | Female | 0.550 (0.189) ** |  |  |  |  |
| RACE | |  |  |  |  |  |
|  | Asian | -0.259 (0.404) | 0.026 (0.092) |  |  |  |
|  | Black | -0.025 (0.554) | 0.136 (0.148) |  |  |  |
|  | Hispanic | 0.416 (0.428) | -0.022 (0.110) |  |  |  |
| FUNDING | |  |  |  |  |  |
|  | Employer | -2.148 (0.206) *** | -0.023 (0.402) | 0.085 (0.585) | 0.229 (0.784) | 0.342 (0.790) |
|  | Fellowship | -0.293 (0.038) *** | 0.080 (0.061) | 0.065 (0.118) | 0.106 (0.177) | -0.030 (0.128) |
|  | Personal | -0.015 (0.047) | -0.017 (0.091) | -0.233 (0.213) | -0.746 (0.351) * | -0.156 (0.218) |
|  | GTA | 0.386 (0.030) *** | -0.076 (0.056) | -0.092 (0.109) | 0.092 (0.194) | -0.071 (0.137) |
| FAMILY | |  |  |  |  |  |
|  | Married | -0.368 (0.026) *** | 0.456 (0.046) *** | 0.231 (0.096) | 0.081 (0.156) | 0.192 (0.106) |
|  | Dependents | -0.213 (0.018) *** | 0.109 (0.035) ** | 0.000 (0.072) | 0.164 (0.086) | -0.081 (0.071) |
| * p<0.1; ** p<0.01; p<0.001 | | | | | | |

| **Table B4. Logistic Regression Coefficients and Standard Errors of the Main Effects Model and Filtered Post-2000 Model for Biological Sciences, Engineering, and Physical Sciences** | | | | | | | |
| --- | --- | --- | --- | --- | --- | --- | --- |
|  | | Biological Sciences | | Engineering | | Physical Sciences | |
|  | Intercept | -2.747 ***  (0.129) | -2.413 ***  (0.134) | -2.444 ***  (0.247) | -2.196 ***  (0.249) | -8.879  (43.95) | 2.028 ***  (0.320) |
| GENDER (ref: male) | |  |  |  |  |  |  |
|  | Female | 0.175 ***  (0.018) | 0.293 ***  (0.020) | 0.186 ***  (0.028) | 0.324 ***  (0.027) | 0.068 **  (0.025) | 0.111 ***  (0.029) |
| RACE (ref: White) | |  |  |  |  |  |  |
|  | Asian | 0.096 *  (0.044) | 0.316 ***  (0.029) | 0.362 ***  (0.035) | 0.440 ***  (0.031) | 0.338 ***  (0.054) | 0.462 ***  (0.045) |
|  | Black | 0.158 *  (0.080) | 0.322 ***  (0.053) | 0.397 ***  (0.069) | 0.390 ***  (0.060) | 0.189 *  (0.096) | 0.327 ***  (0.081) |
|  | Hispanic | 0.017  (0.058) | 0.175 ***  (0.041) | 0.201 ***  (0.059) | 0.251 ***  (0.053) | 0.245 ***  (0.066) | 0.236 ***  (0.063) |
| FUNDING (ref: GRA) | |  |  |  |  |  |  |
|  | Employer | -0.823 ***  (0.085) | -0.971 ***  (0.097) | -1.521 ***  (0.067) | -1.527 ***  (0.079) | -2.124 ***  (0.168) | -2.066 ***  (0.207) |
|  | Fellowship | -0.074 ***  (0.021) | -0.245 ***  (0.024) | -0.193 ***  (0.025) | -0.182 ***  (0.029) | -0.258 ***  (0.029) | -0.254 ***  (0.036) |
|  | Personal | 0.163 ***  (0.029) | -0.096 *  (0.045) | -0.122 ***  (0.034) | -0.163 ***  (0.048) | -0.060  (0.040) | -0.100  (0.070) |
|  | GTA | 0.431 ***  (0.025) | 0.260 ***  (0.035) | 0.272 ***  (0.037) | 0.237 ***  (0.045) | 0.361 ***  (0.026) | 0.317 ***  (0.035) |
| MARITAL (Ref: Not married) | |  |  |  |  |  |  |
|  | Married | -0.115 ***  (0.017) | -0.123 ***  (0.021) | -0.333 ***  (0.021) | -0.260 ***  (0.027) | -0.222 ***  (0.021) | -0.138 ***  (0.029) |
| Dependents (number) | | -0.118 ***  (0.011) | -0.063 ***  (0.016) | -0.161 ***  (0.013) | -0.094 ***  (0.016) | -0.202 ***  (0.015) | -0.157 ***  (0.023) |
|  |  |  |  |  |  |  |  |
|  | Respondents | 116,228 | 68,616 | 88,236 | 49,495 | 77,549 | 38,205 |
|  | Year FE | X | X | X | X | X | X |
|  | Control Variables^ | X | X | X | X | X | X |
|  | Inst Filter (n>30) | X | X | X | X | X | X |
|  | Inst Dummy | X | X | X | X | X | X |
|  | Year, 2001 to 2016 |  | X |  | X |  | X |
| * p<0.1; ** p<0.01; p<0.001  ^ Control variables are Age at Doctorate, Father’s Education, Mother’s Education, and Time to Degree | | | | | | | |

| **Table B5. Main Effects Model with All Field Categories.** | | | | |
| --- | --- | --- | --- | --- |
|  | | Main Effects | Field Interactions (Ref: Biology) | |
|  |  |  | Engineering | Physical Sciences |
| Intercept | | -8.684 (26.66) |  |  |
| GENDER (Ref: Male) | |  |  |  |
|  | Female | 0.225 (0.016) *** | 0.014 (0.028) | -0.127 (0.027) *** |
| RACE (Ref: White) | |  |  |  |
|  | Asian | 0.340 (0.024) *** | 0.154 (0.033) *** | 0.170 (0.039) *** |
|  | Black | 0.349 (0.042) *** | 0.099 (0.064) | 0.123 (0.074) |
|  | Hispanic | 0.166 (0.036) *** | 0.144 (0.057) * | 0.181 (0.061) ** |
| FUNDING (Ref: GRA) | |  |  |  |
|  | Employer | -0.797 (0.082) *** | -0.706 (0.101) *** | -0.912 (0.169) *** |
|  | Fellowship | -0.105 (0.019) *** | -0.107 (0.030) *** | -0.147 (0.034) *** |
|  | Personal | 0.105 (0.028) *** | -0.139 (0.040) *** | 0.015 (0.047) |
|  | GTA | 0.453 (0.024) *** | -0.084 (0.040) * | 0.037 (0.034) |
| FAMILY | |  |  |  |
|  | Married | -0.089 (0.017) *** | -0.218 (0.027) *** | -0.129 (0.028) *** |
|  | Dependents | -0.087 (0.012) *** | -0.039 (0.017) * | -0.013 (0.019) |
| * p<0.1; ** p<0.01; p<0.001  ^ Control variables are Age at Doctorate, Father’s Education, Mother’s Education, and Time to Degree | | | | |

| **Table B6: Percent Unemployed Based on SDR Data (three years post-graduation)** | | | | |
| --- | --- | --- | --- | --- |
| Employment Status at the Time of Graduation | ALL | Biology | Engineering | Physical Sciences |
| SED: Seeking, but No Offer | 8.9% | 10.1% | 7.5% | 8.7% |
| SED: Have offer or not Seeking | 4.1% | 6.0% | 2.4% | 3.3% |
| **Earned PhD since 2001** |  |  |  |  |
| SED: Seeking, but No Offer | 10.0% | 10.0% | 10.4% | 9.7% |
| SED: Have offer or not Seeking | 4.3% | 5.7% | 2.8% | 3.5% |

| **Table B7: Frequency and Percentages of the Reasons for Not Working in a Related Field by Gender and Marital Status for SED Respondents Seeking a Job, But with No Offer** | | | | |
| --- | --- | --- | --- | --- |
| MARITAL STATUS/  REASON FOR NOT WORKING IN RELATED FIELD | MALE | | FEMALE | |
| Married | 142 | | 79 | |
| Not Married | 99 | | 57 | |
| **Pay, Promotion** |  |  |  |  |
| Married | 84 | 59.2% | 35 | 44.3% |
| Not Married | 49 | 34.5% | 27 | 47.4% |
| **Working Condition** |  |  |  |  |
| Married | 55 | 38.7% | 39 | 49.4% |
| Not Married | 33 | 23.2% | 30 | 52.6% |
| **Job Location** |  |  |  |  |
| Married | 72 | 50.7% | 57 | 72.2% |
| Not Married | 46 | 32.4% | 30 | 52.6% |
| **Change in Interest** |  |  |  |  |
| Married | 61 | 43.0% | 41 | 51.9% |
| Not Married | 48 | 33.8% | 31 | 54.4% |
| **Family** |  |  |  |  |
| Married | 42 | 29.6% | 40 | 50.6% |
| Not Married | 15 | 10.6% | 7 | 12.3% |
| **Job Not Avail** |  |  |  |  |
| Married | 96 | 67.6% | 38 | 48.1% |
| Not Married | 62 | 43.7% | 30 | 52.6% |
| **Other** |  |  |  |  |
| Married | 17 | 12.0% | 3 | 3.8% |
| Not Married | 16 | 11.3% | 3 | 5.3% |
